# A randomized trial of over‐the‐counter hearing aids for adults with cognitive decline due to Alzheimer’s Disease and related dementias

**DOI:** 10.1002/alz.095502

**Published:** 2025-01-09

**Authors:** Kimberly D Mueller, Nathaniel A. Chin, Victoria J. Williams, Courtney B. McAlister, Pamela E. Souza

**Affiliations:** ^1^ School of Medicine and Public Health, University of Wisconsin‐Madison, Madison, WI USA; ^2^ Department of Communication Sciences and Disorders, University of Wisconsin‐Madison, Madison, WI USA; ^3^ Wisconsin Registry for Alzheimer’s Prevention, Madison, WI USA; ^4^ Department of Medicine, University of Wisconsin‐Madison School of Medicine and Public Health, Madison, WI USA; ^5^ Wisconsin Alzheimer’s Disease Research Center, Madison, WI USA; ^6^ University of Wisconsin‐Madison, Madison, WI USA; ^7^ Mayo Clinic College of Medicine and Science, La Crosse, WI USA; ^8^ Northwestern University, Evanston, IL USA

## Abstract

**Background:**

Hearing loss occurs commonly with age and is more prevalent among adults with Mild Cognitive Impairment (MCI) or Alzheimer’s Disease and related dementias (ADRD) than among older adults with typical cognitive abilities. Over‐the‐counter (OTC) hearing aids are a recently approved, lower‐cost method of hearing treatment which can be accessed directly by patients. However, there are almost no data which can guide whether to recommend this treatment for adults with MCI or ADRD. OTC aids also have technology constraints that may limit their benefit for wearers with ADRD, who have specific communication and rehabilitation needs.

**Method:**

Participants in this randomized clinical trial will be recruited at the time of diagnosis of either MCI or early ADRD. The trial design embeds a brief hearing assessment within the structure of a memory assessment at Geriatrics and Neurology clinics, in both urban (Madison, WI) and rural (La Crosse, WI) settings. Trial participants will be randomized to receive either OTC aids or communication strategies tailored for adults with cognitive decline. To maximize access for patients (including patients from rural or underserved areas), treatment will be delivered via telehealth. Outcomes will be measured at baseline and after one month of use of the OTC hearing aids or communication strategies. The primary outcome is subjective assessment of dyadic communication by the listener’s primary communication partner. Additional outcomes include hearing‐related quality of life and a novel assessment of communication ability using structured conversation analysis.

**Result:**

This presentation will describe the trial design, expertise and roles of the multidisciplinary study team, mechanism of treatment delivery via telehealth, and planned outcome measurements.

**Conclusion:**

Results of this trial will provide important evidence to guide treatment recommendations in community‐dwelling older adults with MCI or early ADRD. This project will also establish feasibility of conducting hearing assessment as part of a memory assessment.

